# Using a Novel Microfabricated Model of the Alveolar-Capillary Barrier to Investigate the Effect of Matrix Structure on Atelectrauma

**DOI:** 10.1038/s41598-017-12044-9

**Published:** 2017-09-14

**Authors:** N. Higuita-Castro, M. T. Nelson, V. Shukla, P. A. Agudelo-Garcia, W. Zhang, S. M. Duarte-Sanmiguel, J. A. Englert, J. J. Lannutti, D. J. Hansford, S. N. Ghadiali

**Affiliations:** 1 Biomedical Engineering Department, The Ohio State University, Columbus, Ohio, United States; 2 Dorothy M. Davis Heart and Lung Research Institute, The Ohio State University Wexner Medical Center, Columbus, Ohio, United States; 3 Department of Molecular and Cellular Biochemistry, The Ohio State University, Columbus, Ohio, United States; 4 Department of Internal Medicine, Division of Pulmonary, Critical Care and Sleep Medicine, The Ohio State University Wexner Medical Center, Columbus, Ohio, United States; 5 Human Nutrition Program, The Ohio State University, Columbus, Ohio, United States; 6 Department of Material Sciences and Engineering, The Ohio State University, Columbus, Ohio, United States

## Abstract

The alveolar-capillary barrier is composed of epithelial and endothelial cells interacting across a fibrous extracelluar matrix (ECM). Although remodeling of the ECM occurs during several lung disorders, it is not known how fiber structure and mechanics influences cell injury during cyclic airway reopening as occurs during mechanical ventilation (atelectrauma). We have developed a novel *in vitro* platform that mimics the micro/nano-scale architecture of the alveolar microenvironment and have used this system to investigate how ECM microstructural properties influence epithelial cell injury during airway reopening. In addition to epithelial-endothelial interactions, our platform accounts for the fibrous topography of the basal membrane and allows for easy modulation of fiber size/diameter, density and stiffness. Results indicate that fiber stiffness and topography significantly influence epithelial/endothelial barrier function where increased fiber stiffness/density resulted in altered cytoskeletal structure, increased tight junction (TJ) formation and reduced barrier permeability. However, cells on rigid/dense fibers were also more susceptible to injury during airway reopening. These results indicate that changes in the mechanics and architecture of the lung microenvironment can significantly alter cell function and injury and demonstrate the importance of implementing *in vitro* models that more closely resemble the natural conditions of the lung microenvironment.

## Introduction

Mechanical Ventilation (MV) is one of the most effective strategies to provide supportive care to patients suffering from Acute Lung Injury (ALI) and/or the Acute Respiratory Distress Syndrome (ARDS)^1^. Although critical for patient survival, MV may lead to mechanically-induced tissue damage and significant local and systemic inflammation^1, 2^. Even when protective MV strategies, such as low-tidal volume and patient-tailored positive end-expiratory pressure (PEEP) are used, ALI and ARDS patients are still susceptible to Ventilator-induced Lung Injury (VILI). 5–15% of ventilated patients will develop VILI, which has a mortality rate of 34–60%^3^. During the positive-pressure ventilation of injured lungs, several mechanical factors can exacerbate the existing lung injury. For example, the overdistension of lung tissue during high tidal volume ventilation can cause cellular injury and disruption of the alveolar-capillary barrier, known as volutrauma^4, 5^. In addition, the high transmural pressures exerted on the lung can lead to the release of pro-inflammatory cytokines into the circulation^6, 7^ and this may lead to multiple-system organ failure^8^. Finally, the cyclic collapse and reopening of fluid occluded lung regions can result in a form of lung injury known as atelectrauma. During atelectrauma, the surface tension forces generated during the reopening of fluid-occluded airway/alveoli can cause significant epithelial cell injury/death, cell detachment and disruption of the alveolar-capillary barrier^9^. Interestingly, recent *in vivo* and *in vitro* studies indicate that although atelectrauma may not induce a large inflammatory response^10^, it is the main source of cellular injury and barrier disruption and is more damaging that volutrauma^11, 12^.

Although fundamental research has been carried out to elucidate the mechanisms responsible for cell injury during cyclic stretching and the reopening of fluid-filled airways, the effect of the lung extracellular matrix (ECM) structure and intrinsic epithelial-endothelial interactions on the alveolar-capillary barrier have not been fully explored. Specifically, previous studies have investigated how different mechanical stresses, including shear stress, cyclic stretching and surface tension forces alter lung epithelial or endothelial cell viability and behavior during mechanical ventilation^5, 13–21^. In these studies, cells are typically cultured on non-porous flat substrates and injury responses are monitored for monocultures of either epithelial or endothelial cells. For example, the Ghadiali and Gaver groups have used parallel-plate perfusion chambers to expose epithelial cells cultured on a rigid glass substrate to propagating microbubbles of air to simulate the reopening of occluded airways^13–16^. These authors demonstrated that structural characteristics of the airways (i.e. airway diameter) and reopening velocity influence the degree of cell injury during airway reopening where cells in smaller airways and/or exposed to slow reopening velocities are highly susceptible to plasma membrane rupture and cell detachment. These studies also confirmed the protective effect of pulmonary surfactant and actin cytoskeleton de-polymerizating agents. Recently, we used similar techniques to demonstrate that clinically approved drugs, i.e. Simvastatin, can be used to prevent cell injury during reopening^22^. Tschumperlin and collaborators pioneered the use of cell stretching devices to model over-distension injury where primary lung epithelial cells are cultured on a flexible non-porous membrane and exposed to ~12–50% equibiaxial strain^21^. These authors demonstrated that the magnitude of cell injury was proportional to the magnitude of deformation while others have used similar systems to demonstrate how inhibiting lipid-trafficking can increase the amount of cell injury^23, 24^. For endothelial cells, Birukov *et al*. reported the use of gelatin-coated glass substrates with pulmonary endothelial cell monolayers that were exposed to laminar shear stress (10 dynes/cm^2^)^18^. In this model, shear stress induced significant changes in actin cytoskeletal organization, with increased stress fiber formation and transient peripheral translocation of cortactin through a Rac GTPase-dependent pathway^18^. Birukov and collaborators also evaluated the effect of cyclic stretching on the pulmonary endothelial barrier properties, using human pulmonary endothelial cells cultured on flexible non-porous collagen-coated substrates^19^. In this study, the authors described how cyclic stretching and/or thrombin exposure increases gap formation and barrier deregulation^19^.

Although these studies have provided invaluable insights into the pathology of VILI, these previous *in vitro* models do not accurately simulate the physical and structural properties of the lung microenvironment and ECM which contains a compliant fiber architecture. In addition, to our knowledge, previous *in vitro* models of cell injury during VILI did not account for the epithelial/basement-membrane/endothelial structure that exists *in vivo*. Developing *in vitro* models that accurately simulate the alveolar-capillary barrier of the lung is fundamental to achieve a better understanding of the mechanisms involved in pathophysiological conditions such as VILI. Previous reports have demonstrated the applicability of these types of biomimetic microsystems to conduct lung-nanotoxicology studies, their potential application for drug screening, and modeling of a wide range of lung inflammatory disorders^25–27^. However, these “lung-on-a-chip” (LOC) systems do not account for the complex nature of the lung ECM that consists of a compliant fibrous matrix and/or model the changes in the matrix that can occur during several lung disorders. For example, pulmonary fibrosis involves significant changes in ECM properties, i.e. fiber density and stiffness, and therefore enhanced “lung-on-a-chip” technologies that account for these changes would increase their clinical relevance. Furthermore, the previous LOCs were not designed to evaluate how the mechanical forces generated during MV alter alveolar-capillary barrier function or cell injury. Therefore, the goal of this study was to develop a novel micro-nano biomimetic system that more closely resembles the alveolar-capillary microenvironment by allowing the co-culture of epithelial and endothelial cells on a compliant fibrous polymeric substrate, which resembles the lung's basal lamina structure. Since we hypothesize that changes in the alveolar microenvironment may influence the degree of cell injury during airway reopening, we have used our novel micro-nano biomimetic system to investigate how ECM structure/mechanics influence alveolar-capillary barrier properties and epithelial cell injury during airway reopening.

## Results

### Micro-nano device fabrication

**Figure 1:**
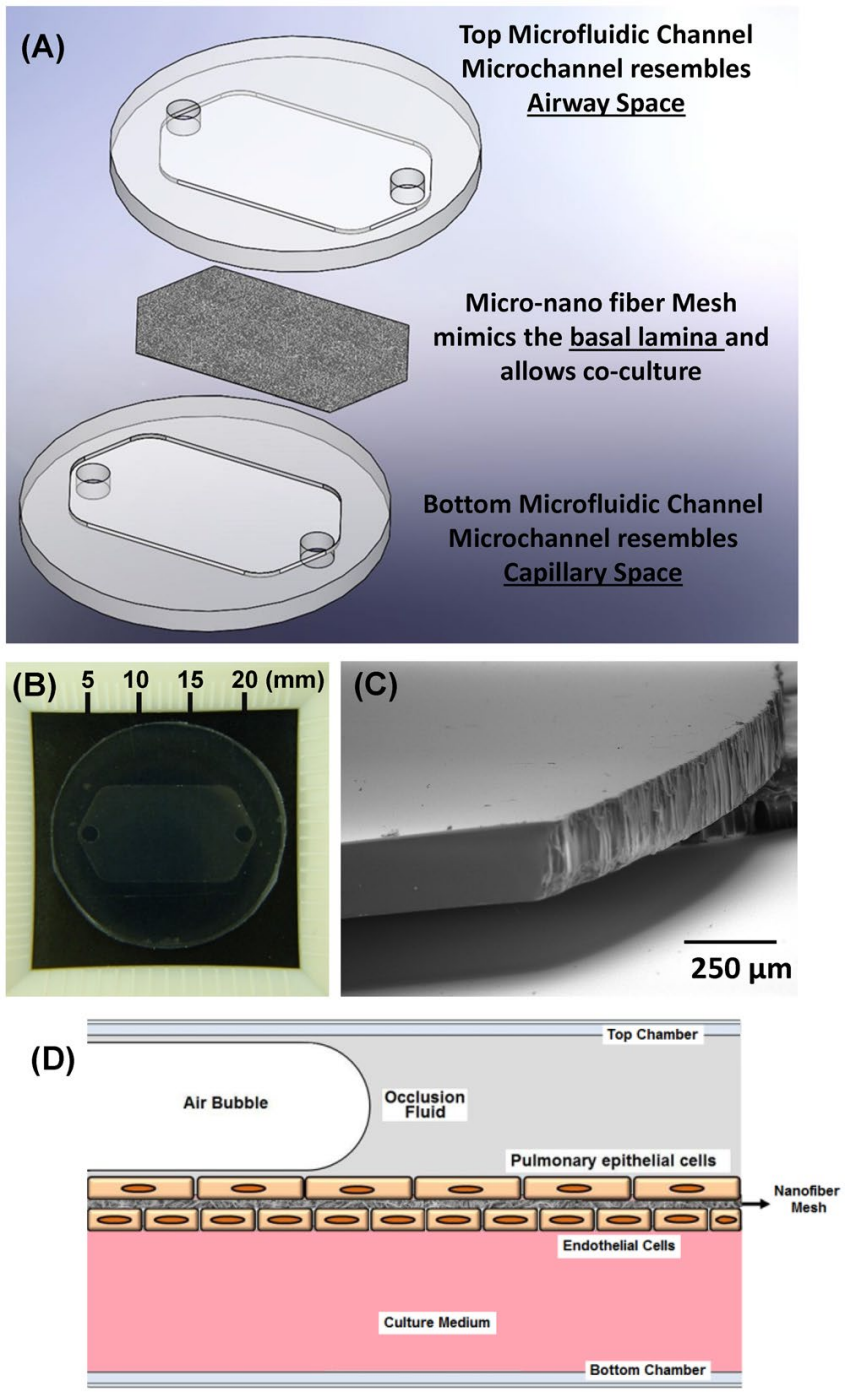
(**A**) Schematic representation of the micro-nano biomimetic system used in this study. (**B**) Image of PDMS microfluidic chamber, (**C**) SEM image of SU8-2075 spin coated photoresist layer with an average thickness of 200 µm, and (**D**) schematic representation of occluded airway reopening simulation for epithelia/endothelial cells co-cultured on opposite sides of the fiber meshes.

**Figure 2:**
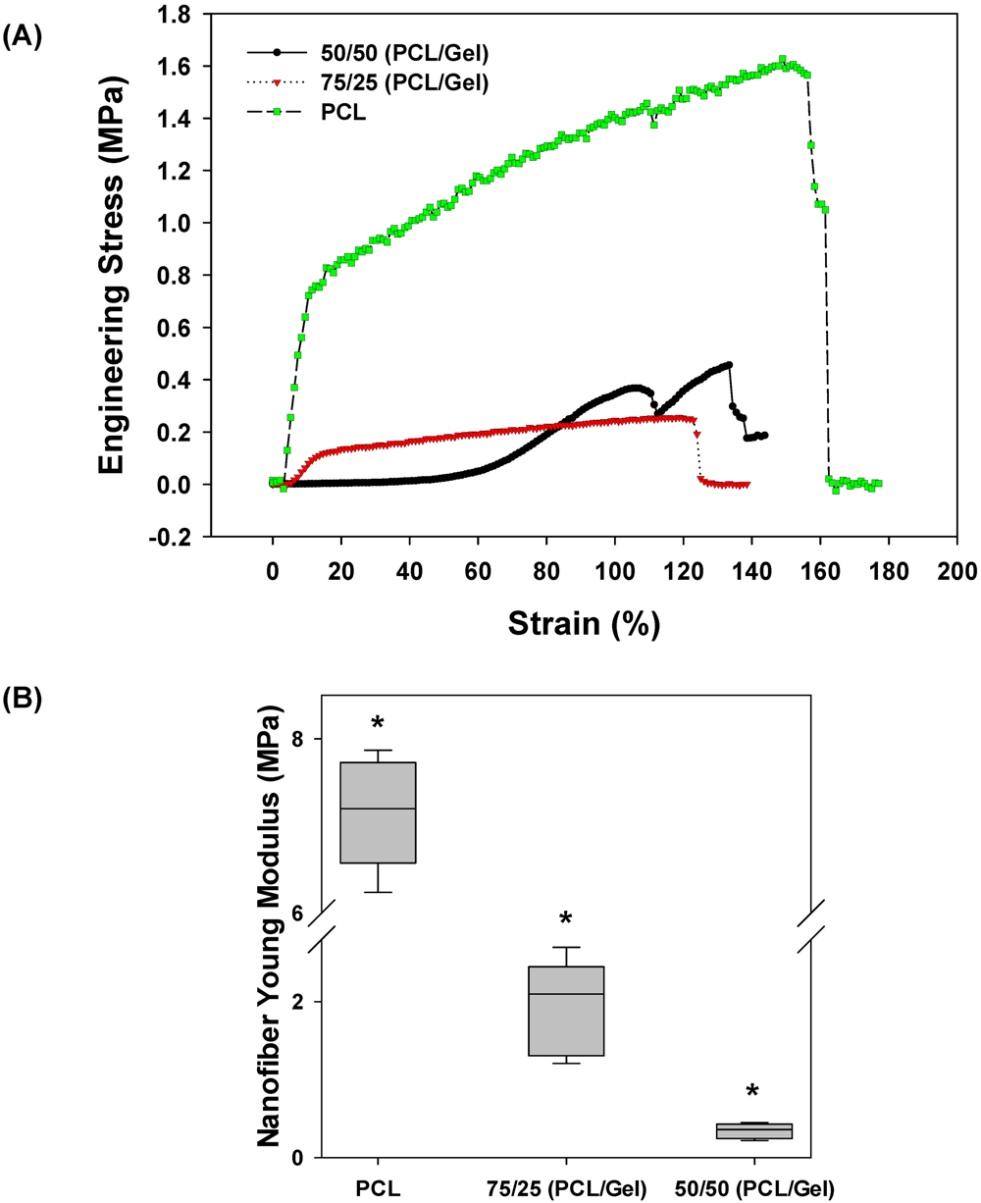
Tensile test results for wet nano fiber meshes prepared using different PCL-to-gelatin rations. (**A**) Representative stress vs. strain curves for the different fiber mesh compositions used in this study and (**B**) Computed Young Modulus values. *Indicates significant difference between groups (p-value ≤ 0.009).

**Figure 3:**
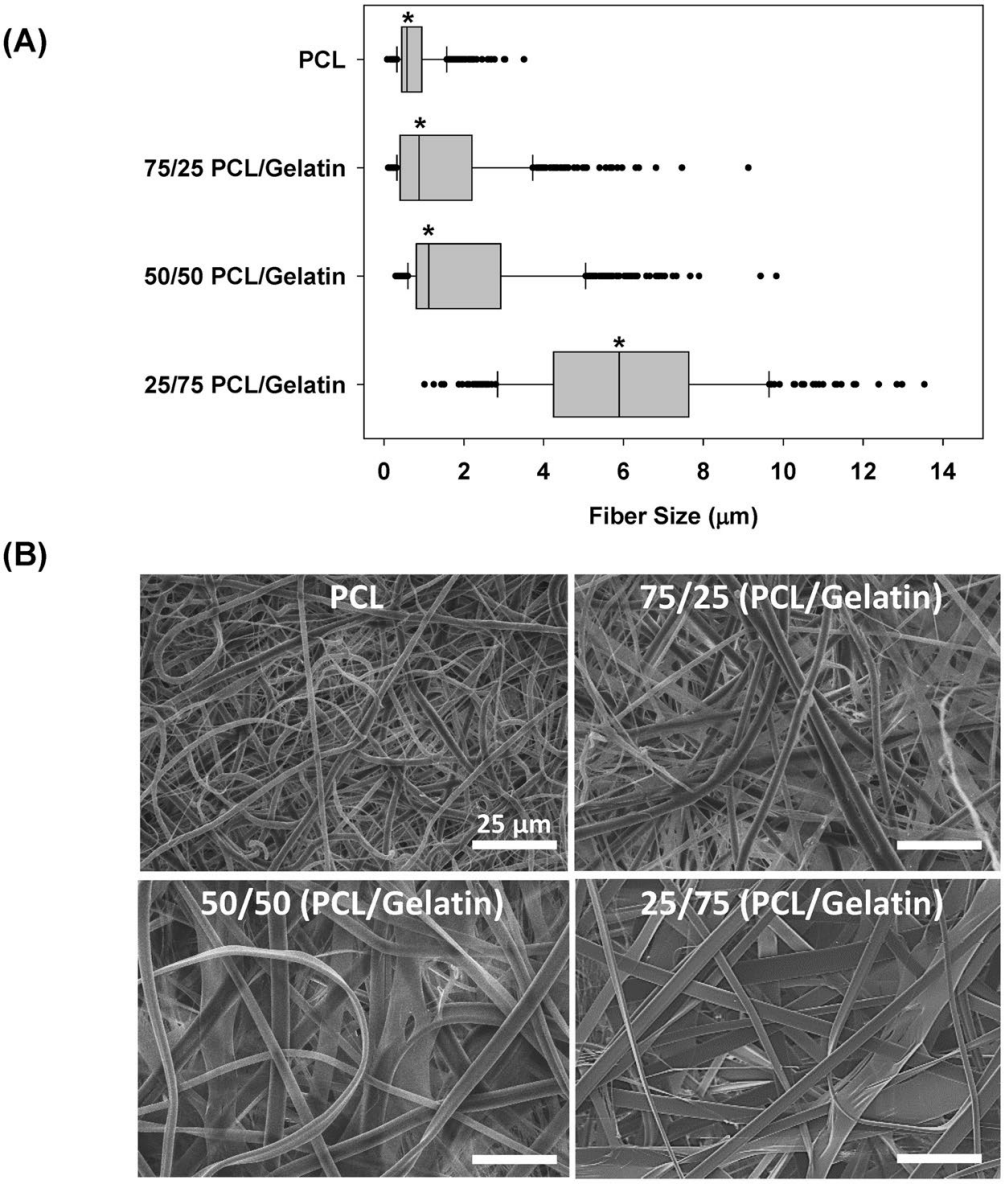
(**A**) Fiber size distribution for fibers fabricated with different PCL/Gelatin ratios, and (**B**) SEM images showing detail of fiber architecture of representative fiber groups (scale bars: 25 µm). *Indicates significant difference with respect to other groups (p-value < 0.001).

A schematic representation of the micro-nano biomimetic system used in this study is shown in Fig. 1A and the dimensions of the device are shown in Fig. 1B,C. The microfluidic chambers patterned on the Si wafers had a thickness of 200 µm (Fig. 1C). In this study, four different fiber matrices were made using different ratios of polycaprolactone (PCL) and gelatin and three of these matrices (100% PCL, 75/25 PCL/gelatin and 50/50 PCL/gelatin) were subjected to uniaxial mechanical testing as shown in Fig. 2. Nanofiber stiffness was successfully modulated by varying the polymer blend composition where tensile tests revealed that adding gelatin to the polymer blend significantly modulates the stress-strain relationships for the fiber mesh (Fig. 2A). Specifically, increasing amounts of gelatin reduces the linear elastic modulus of the fiber meshes, with median modulus values of 0.36, 2.10, and 7.20 MPa for pre-wetted fiber meshes fabricated with blends containing 50/50 (PCL/gelatin), 75/25 (PCL/gelatin) and pure PCL, respectively (p ≤ 0.009) (Fig. 2A,B). As shown in Fig. 3A, fiber size increased significantly (p < 0.001), proportional to the gelatin content, with median fiber sizes of 5.9, 1.12, 0.88 and 0.57 µm for 25/75 (PCL/gelatin), 50/50 (PCL/gelatin), 75/25 (PCL/gelatin) and PCL fibers, respectively. As shown in Fig. 3B, fiber morphology also changed depending on the gelatin concentration, where the use of increased gelatin concentrations yield fibers with a ribbon-like structure while increasing PCL concentration leads to a more dense filament-like structure.

### Cell culture

**Figure 4:**
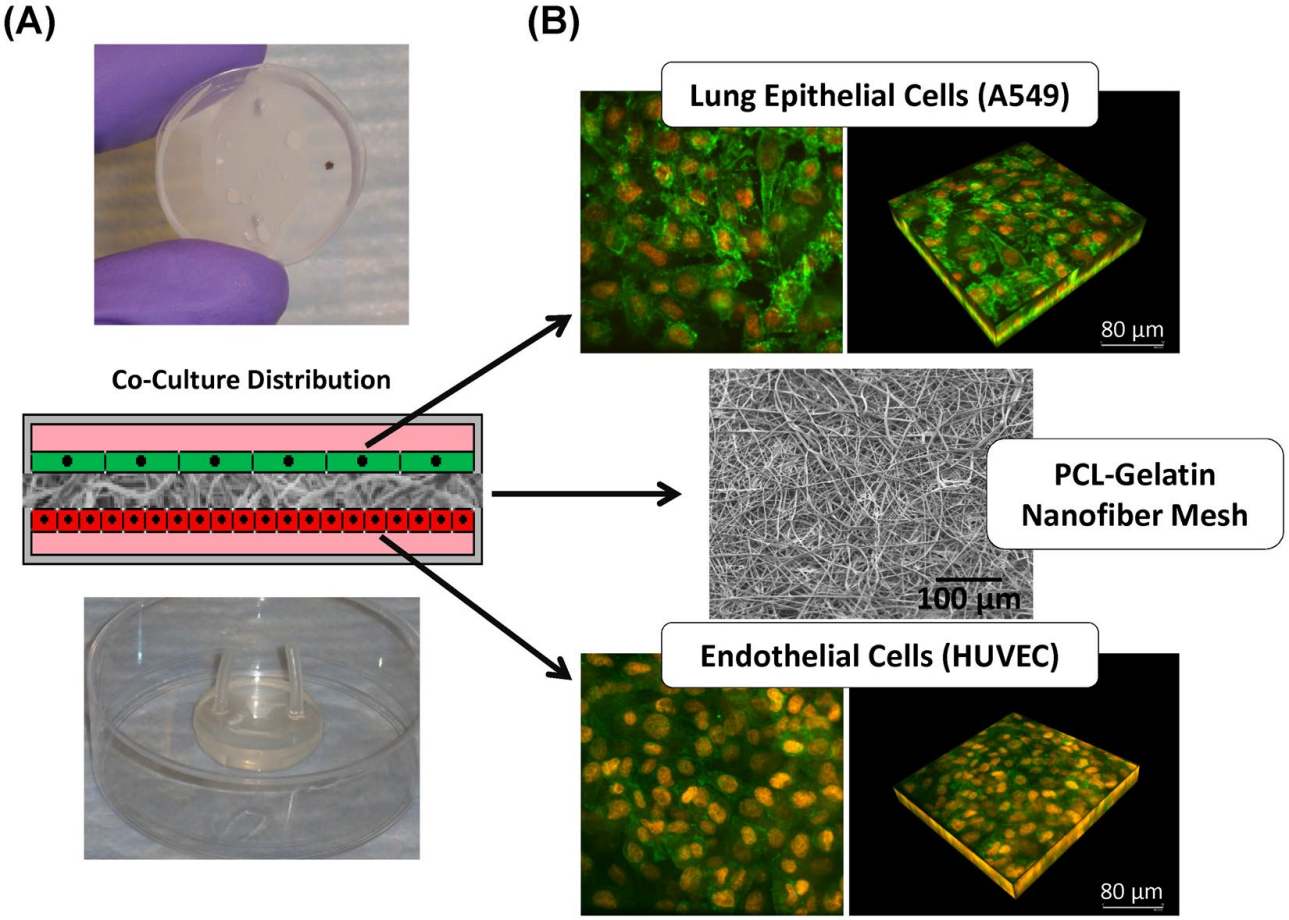
(**A**) Schematic representation of co-culture system and images of assembled micro-nano fluidic device with co-cultured cells, and (**B**) confocal images of epithelial and endothelial cells co-cultured on a 75/25 (PCL/gelatin) fiber mesh (Red: cell nuclei stained with Propidium Iodide (PI)/Rnase, and Green: actin cytoskeleton stained with Alexa Fluor 488 Phalloidin).

Using this system we were able to successfully seed lung epithelial and endothelial cells in a co-culture configuration on ~20 μm thick PCL-gelatin fiber meshes. Figure 4A shows photographs of the micro-nano biomimetic system used in this study, and a diagram illustrating the distribution of the different cell types within the system. Figure 4B shows confocal fluorescence images of the cells (epithelial and endothelial) cultured on the PCL mesh and demonstrate a high degree of confluence. We also show scanning electron microscopy (SEM) images of the micro-nano fibrous substrate used for cell culture in Fig. 4B.

### Evaluation of cellular responses

#### Actin cytoskeleton and Tight junction formation

**Figure 5:**
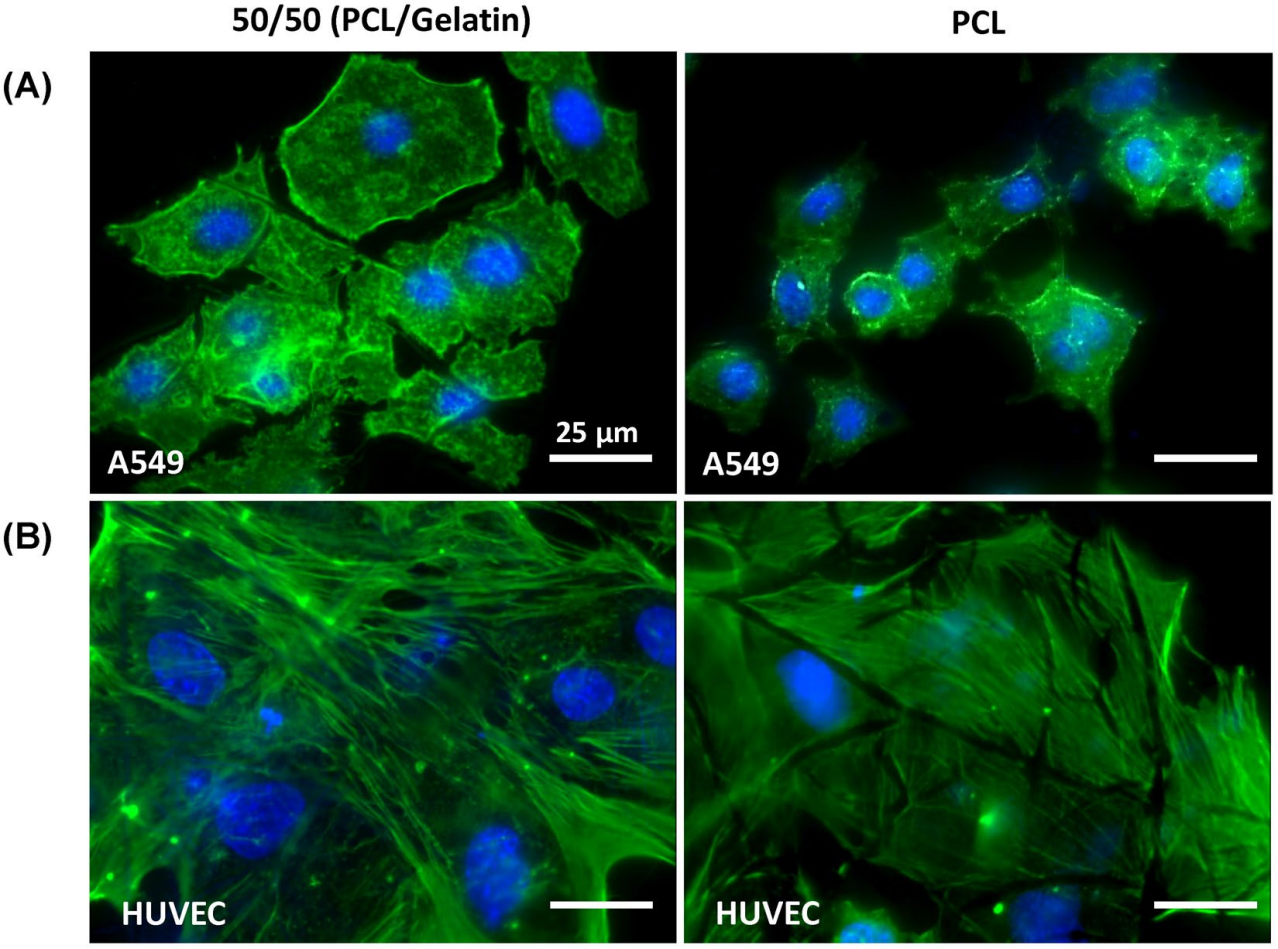
Actin staining (green) and cell nuclei (blue) of (**A**) epithelial (A549) and (**B**) endothelial (HUVEC) cells cultured on PCL (right) or 50/50 (PCL/gelatin) (left) fibers. Scale bar 25 µm.

**Figure 6:**
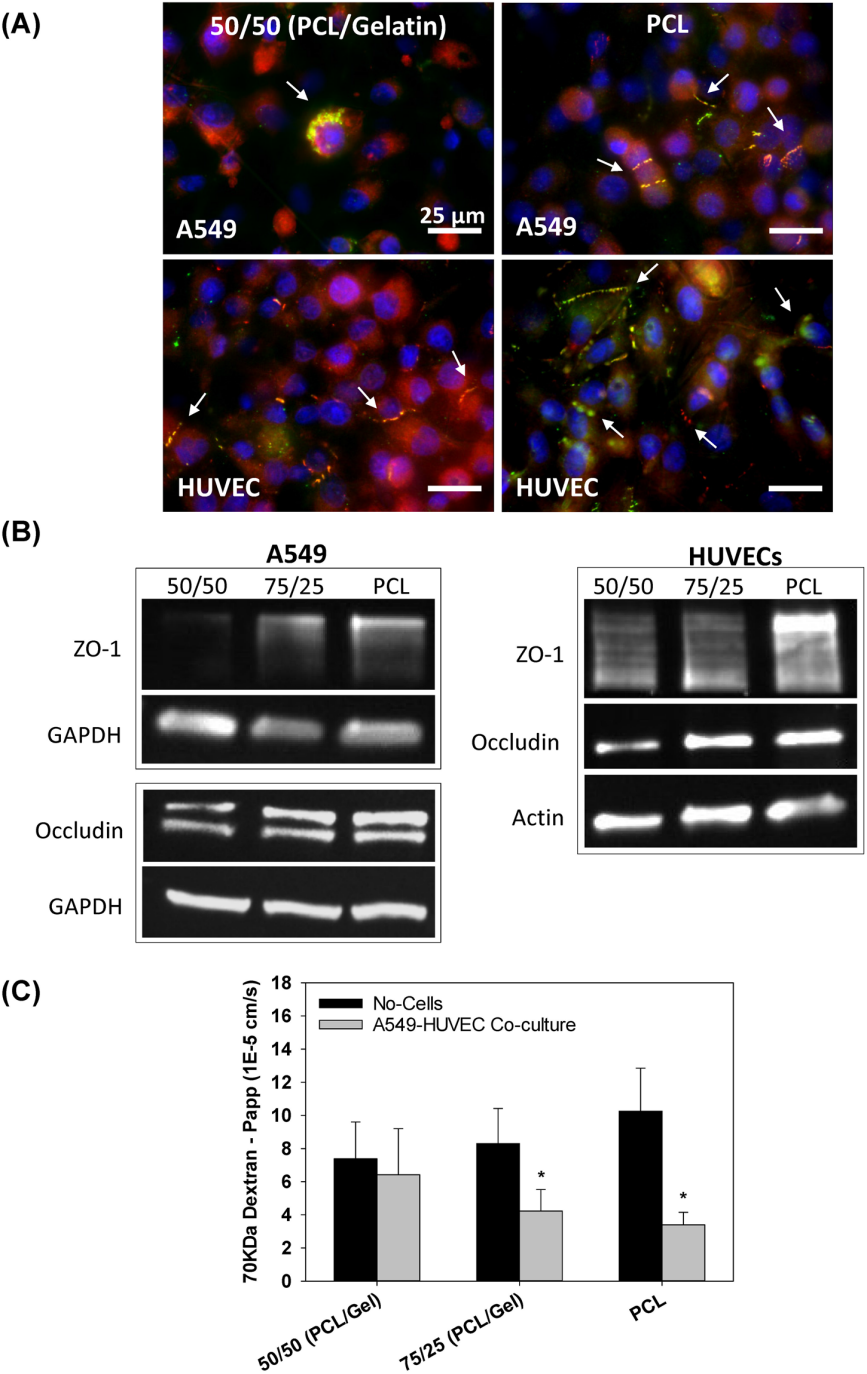
(**A**) tight junctions staining (ZO-1: red, Occludin: green, nuclei: blue) of epithelial and endothelial cells co-cultured on PCL (right) or 50/50 (PCL/gelatin) (left) fibers (scale bars: 25 µm). (**B**) Characterization of ZO-1 and occludin relative expression, and (**C**) transport of 70 KDa FITC-labeled dextran across co-cultured epithelial/endothelial cells after 60 minutes (2-day co-cultures) (mean ± SEM$$)$$. *Indicates significant difference with respect to No-cells (control) group (p =  < 0.029).

As shown in Fig. 5, cells cultured on fiber meshes with different stiffness and structure exhibited different patterns of actin cytoskeletal distribution. Before reaching a confluent state, epithelial cells cultured on softer, less dense fibers (i.e. 50/50 PCL/gelatin fibers), had a more spread morphology in comparison with those on a more rigid, dense fiber mesh (i.e. 100% PCL fibers) (Fig. 5A). Endothelial cells presented spread morphology on both the soft and rigid fiber meshes with increased formation of actin stress fibers when cultured on 50/50 (PCL/gelatin) fibers vs. PCL fibers (Fig. 5B). As shown in Fig. 6A, cells cultured on fiber meshes with different stiffness/structure also exhibited different amounts of epithelial and endothelial tight junctions (ZO-1 and occludin) after 3-days of co-culture. For cells co-cultured on the softer, less-dense 50/50 fibers, we observed little to no ZO-1 or occludin formation in epithelial cells and minimal ZO-1 and occludin formation in endothelial cells. Conversely, for cells co-cultured on the stiffer more-dense PCL fibers, we observed significant ZO-1 and occludin formation in both epithelial and endothelial cells. However, endothelial cells exhibited more defined tight junctions compared to epithelial cells (Fig. 6A). This increase in TJ protein expression on the more rigid/dense PCL fibers was confirmed via western blot analysis of endothelial and epithelial cells cultured on the different fiber substrates (Fig. 6B).

#### Epithelial/endothelial barrier function

A decreasing trend in dextran transport was observed across epithelial/endothelial co-cultures on PCL and 75/25 (PCL/gelatin) fiber meshes in comparison with the 50/50 (PCL/gelatin) fibers (Fig. 6C). Specifically, compared to control conditions with no cells, the apparent permeability coefficient decreased significantly (p < 0.029) by 49% and 67% for cells co-cultured on 75/25 (PCL/gelatin) and 100% PCL fibers. In contrast, cells cultured on the 50/50 (PCL/gelatin) fibers did not provide significant barrier function since these cells exhibited an apparent permeability that was not statistically different than the control, no-cell conditions (p = 0.485).

### Fluid-filled airway reopening simulations

**Figure 7:**
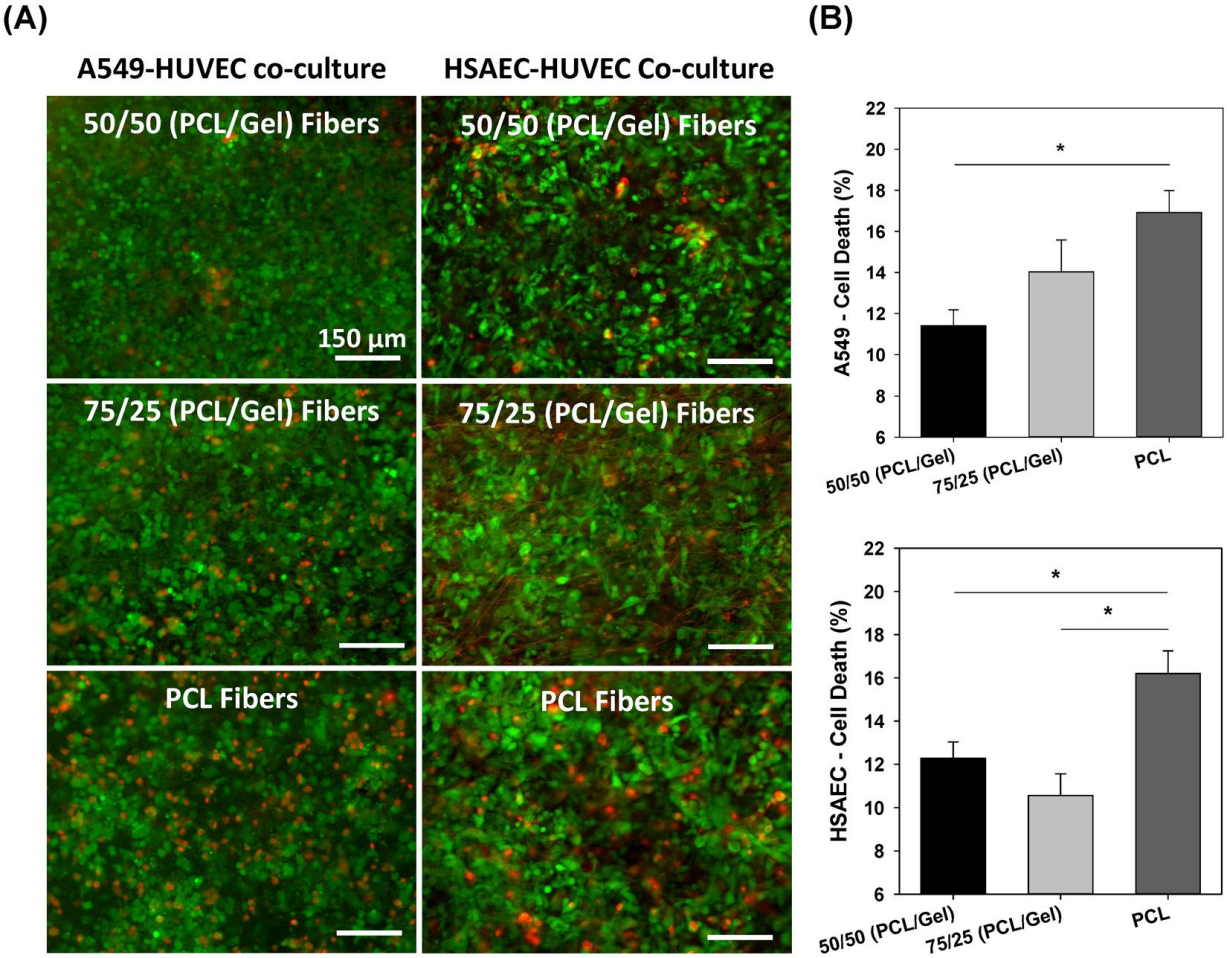
Airway reopening simulations for epithelial/endothelial cells co-cultured on 50/50, 75/25, and 100/0 (PCL/gelatin) fiber meshes after simulation of airway reopening. (**A**) Live/dead fluorescence imaging of both A549 and primary human small airway epithelial cells after 5 reopening events and (**B**) quantification of percentage of cell death after propagation of 5 air bubbles for A549 and primary epithelial cells (mean ± SEM) (scale bars: 150 µm). *Indicates significant difference between groups (p-value ≤ 0.018).

As shown in Fig. 7, simulating 5 airway reopening events resulted in a significantly higher percentage of cellular death for cells cultured on PCL fibers in comparison with those on gelatin-containing fibers (p ≤ 0.018 for cells on PCL vs. 50/50 fibers). After 5 airway reopening events epithelial cell injury values of 11.4%, 14.0%, and 16.9% were observed for A549 and HUVECs co-cultured on matrigel-coated fibers containing 50/50, 75/25, and 100/0 (PCL/gelatin), respectively (Fig. 7A,B). These results were validated by repeating these airway reopening experiments using co-cultures of primary human small airway cells (HSAECs) and HUVECs. For these studies, 5 airway reopening events also resulted in significantly higher cell death for cells culture on PCL fibers (p < 0.02 for cells on PCL vs 50/50 and for cell on PCL vs 75/25 fibers). The mean injury after airway reopening for primary cells cultured on matrigel-coated fibers containing 50/50, 75/25, and 100/0 (PCL/gelatin) were 12.3%, 10.6%, and 16.2%respectively (Fig. 7A,B).

## Discussion

The alveolar-capillary microenvironment is a complex structure that normally provides both efficient gas-exchange and a tight barrier that prevents fluid from entering airspaces within the lung^18^. Disruption of this barrier during acute lung injury leads to pulmonary edema and severe hypoxia and these patients often require mechanical ventilation (MV). Although life-saving, MV generates biophysical forces that can exacerbate lung injury and barrier disruption. Although most investigators have focused on how over-distension causes lung injury^19, 21^, recent studies indicate that the surface tension forces generated during the reopening of atelectatic airways/alveoli is the main source of cellular injury^11, 12^. However, most previous studies have only investigated how airway reopening causes injury to epithelial cells cultured on rigid glass or plastic substrates^13–16^ and therefore did not account for the complex nature of the lung ECM that consists of a compliant fibrous matrix. Recently, our group developed an atelectrauma model that accounted for one aspect of the lung microenvironment where cells were cultured on soft polyacrylamide gels to account for the complaint nature of lung tissue^28^. That study demonstrated that increases in substrate stiffness leads to a change in cell morphology which in turn causes increased cell injury during airway reopening. Interestingly, increased substrate stiffness also led to increased focal adhesion protein expression (i.e. increased expression of phosphorylated-PAX) and this resulted in less cell detachment during airway reopening. Although that study provided novel insights into the effect of lung compliance on atelectrauma, it did not account for the fibrous nature of the lung ECM and could not be used to evaluate epithelial-endothelial cell interactions and barrier permeability. Therefore, in this study we have used electrospinning techniques to model the fibrous nature of the lung ECM and have developed a novel co-culture model of epithelial-endothelial cell interaction that mimics the *in vivo* physiology. We have incorporated this co-culture model into a microfluidic system that can simulate the surface tension forces responsible for cell injury during airway reopening and have used this system to evaluate how ECM structure alters barrier permeability and cell injury.

In this study, electrospinning of PCL/gelatin polymer blends was used to model the ECM since electrospun fibers have been shown to simulate the natural topography of the basal membrane^29–33^. Electrospinning allowed for the precise modulation of fiber mesh properties such as fiber diameter, stiffness and density and as a result, we were able to simulate different micro-architectures relevant to normal and abnormal lung tissue. For example, during pulmonary fibrosis increased collagen deposition and remodeling leads to a dense ECM and a significant increase in local tissue stiffness^34^. On the other hand, emphysema involves destruction of the lung ECM architecture and thus a less dense fiber network. Therefore, as shown in Figs 2 and 3B, in this study the 100% PCL fibers can be considered representative of a fibrotic state while the 50/50 PCL/gelatin fibers can be considered representative of the emphysematic state. Of course the clinical pathology often involves other structural changes such as enlarged airspaces during emphysema. The implementation of soft lithography during the fabrication of the current device allows for precise control over channel morphology and dimensions, allowing for easy modulation of the channel volume and length, and therefore the simulation of airways with different dimensions. Although the current study focused solely on how changes in fiber diameter and density influence barrier function and cell injury, future studies could use this system to investigate how other parameters that change during disease, such as airway dimensions, alter barrier function and atelectrauma.

The use of 100% PCL and 75/25 PCL/gelatin mixture lead to dense fiber meshes and increased stiffness as measured by the linear elastic modulus (Figs 2 and 3). Epithelial and endothelial cells cultured on this rigid dense fiber matrix formed tighter monolayers with higher tight junction formation and more effective barrier properties. In contrast, the polymer blends containing 50/50 PCL/gelatin formed softer, low density fiber meshes, which lead to looser monolayers with lower barrier properties, as measured by FITC-labeled dextran (70 KDa) transport across epithelial/endothelial cell co-cultures (Fig. 6C). This response in terms of better barrier properties for cells cultured on the rigid/dense PCL fibers could be explained by the observed changes in actin cytoskeletal distribution and TJ formation for epithelial and endothelial cells. Endothelial cells on soft 50/50 fibers presented higher formation of actin stress fibers and lower expression of TJs (Figs 5 and 6). As described by Wysolmerski and Lagunoff ^35^, and subsequently by Garcia *et al*.^36^, these type of changes in the actin cytoskeleton of lung endothelial cells play a key role in the regulation of endothelial retraction and barrier dysfunction through a pathway dependent on myosin light chain kinase (MLCK) activation and myosin light chain phosphorylation^35–37^. Interestingly, epithelial cells on the soft 50/50 fibers presented diffuse actin staining while epithelial cells on rigid 100% PCL fibers exhibited peri-nuclear condensation of actin. Given the lack of actin stress fibers in epithelial cells, it is unlikely that epithelial retraction plays a significant role in barrier dysfunction. However, epithelial cells cultured on the rigid 100% PCL fibers did exhibit modest TJ formation and this result is consistent with previous studies where A549 cells cultured at an air-liquid did express ZO-1 and occludin in culture. However, epithelial cells cultured on the soft 50/50 fibers did not exhibit TJ formation and this could also partially explain why the co-culture of epithelial/endothelial cells on these soft fibers did not exhibit decreased permeability.

Although the epithelial-endothelial co-culture presented better barrier properties on rigid PCL fiber meshes, airway reopening simulations indicate that epithelial cell death was significantly higher after airway reopening for cells co-cultured on the highly dense/rigid PCL nanofibers. In contrast, the degree of epithelial cells death was lower for cells co-cultured on the less dense/rigid 75/25 and 50/50 PCL/gelatin meshes (Fig. 7A,B). This result is in agreement with previous work by our group, where lung epithelial cells cultured on a stiffer polyacrylamide gels were more susceptible to plasma membrane rupture during airway reopening in comparison with cells on softer gels^28^. In that study we demonstrated that this differential cellular response to substrate stiffness could be explained by significant changes in cell morphology where cells on softer substrates exhibited a flatter topography. As described by Jacob and Gaver^38^, this flatter morphology dissipates the hydrodynamic forces applied by the moving air-liquid interface and is therefore consistent with less cell injury during reopening. In line with these previous studies, in this study epithelial cells on softer 50/50 fibers presented a more spread morphology in comparison with the rounded cells observed on the rigid fibers (Fig. 5A). In addition to responding to matrix stiffness, in this case we also hypothesized decreased spreading on the 100% PCL fibers could be attributed, in part, to a higher density per unit area of submicron-scale fiber structures (characteristic of the rigid PCL fiber meshes) with which the cells interact strongly and would limit their spreading. However, we acknowledge that there are other potential explanations for higher cell injury on the stiffer fibers. First, rigid fiber meshes will sustain less deformation during reopening and as a result, the forces applied by the moving air-liquid interface may be “absorbed” by the epithelial cells resulting in more cell deformation and a higher chance of plasma membrane rupture. An alternative hypothesis is that higher TJ expression in epithelial cells on the rigid PCL fibers may alter the way hydrodynamic stresses are distributed within the cell. In fact, a study by Gaver’s group indicates that higher expression of the cytoplasmic TJ multiprotein ZO-1, which directly interact with actin filaments, provides enhanced structural support to cells, dampening out the tangential component of the pressure gradient imposed during airway reopening^39^. It is also important to consider the possible effect of TJ formation in the response of epithelial cells during airway reopening, since the higher expression of cytoplasmic TJs (cell-cell adhesion points) for epithelial cells on rigid PCL fibers may alter stress distribution within the monolayer and thus the strains experienced by the plasma membrane. However, future computational studies that simulate cell deformation during airway reopening may be needed to identify the role of TJ on cell injury^40, 41^.

Although we have developed a novel system that simulates important biophysical and physiological features of the lung microenvironment, including ECM fiber architecture, endothelial-epithelial interactions and the fluid mechanical forces responsible for atelectasis, future studies could incorporate additional features to more accurately mimic the lung microenvironment. First, this study utilized non-biological polymers due to the ease and controllability of these systems. However, future studies could incorporate electrospun biopolymers such as elastin and collagen^42^ and could also introduce interstitial cells such as lung fibroblasts which could remodel the extracellular matrix. In this study, we used our system to investigate how one type of mechanical force, surface tension forces during airway reopening (i.e. atelectrauma), influences cellular responses. However, other mechanical forces such as mechanical stretch, may also occur in mechanically ventilated lungs. Although the current PCL system is not particularly amenable to simulating mechanical stretching during volutrama due to the very stiff nature of this polymer, future studies could use other more compliant polymers which would allow for the simultaneous application of stretching and surface tension forces. Although the current study indicates that ECM mechanics may be an important determinate of ventilation induced lung injury, future studies should test predictions from the *in vitro* system using *in vivo* models. For example, future studies could use mouse models of emphysema and fibrosis to directly test the hypothesis that ventilation of rigid lungs during fibrosis would lead to more cell injury while ventilation of more compliant lungs would lead to more barrier disruption. Finally, although the current device accounts for epithelial-endothelial interactions, future studies could incorporate other cells that play an important role in the alveolar microenvironment such as alveolar macrophages.

## Conclusions

In summary, we have developed a micro-nano biomimetic device that more closely replicates the alveolar-capillary barrier and have used this system to investigate how ECM mechanics and structure alters barrier permeability and cell injury during airway reopening. This system accounted for epithelial-endothelial interactions present at the alveolar level, the natural fibrous topography of the basal membrane, and allowed for easy modulation of extracellular matrix stiffness/structure. Using this device, we found that epithelial and endothelial cells are responsive to changes in fiber stiffness and architecture, where epithelial cells developed a more spread morphology and exhibit lower TJ formation on softer and less dense fiber meshes. Endothelial cells presented a similar response in terms of TJ formation but had increased formation of actin stress fibers on softer fibers. Endothelial cells also presented a fairly constant spread morphology on both soft and rigid fiber meshes. These responses lead to decreased barrier permeability for cells co-cultured on rigid and denser fiber meshes. Changes in substrate stiffness and architecture also influenced the degree of cell injury during airway reopening, where cells co-cultured on dense, rigid fiber meshes were more susceptible to injury presumably due to changes in cell morphology observed for epithelial cells cultured on rigid vs. soft fiber meshes. These results demonstrate the importance of implementing *in vitro* models that more closely resemble the natural conditions of the lung microenvironment. Continued application of this system could lead to a cost-effective, controlled, and systematic analysis of the biomechanical mechanisms involved in ventilator induced lung injury as well as several other pulmonary pathologies.

## Materials and Methods

### Micro-nano device fabrication

The system developed in this study consists of a three-dimensional multilayered structure (Fig. 1A) that contains two microfluidic chambers which model the airway space and capillary lumen. These chambers are interfaced with a porous mesh of polymeric fibers, which mimics the basal lamina in the lung and is the substrate for co-culturing lung epithelial and endothelial cells. The top and bottom chambers of the device were fabricated using soft lithography techniques and are made of polydimethylsiloxane (PDMS), while the fiber mesh was obtained via electrospinning of a polymer blend containing polycaprolactone – (PCL) and gelatin (porcine skin gelatin type A).

#### Microfluidic chambers

The layout of the two microfluidic chambers was created using the SolidWorks 3D CAD Design Software (Dassault Systèmes SolidWorks Corp., Waltham, MA) and then printed on a transparency for subsequent use as a mask during the photolithography process. Both chambers were fabricated in PDMS (Dow Corning, Midland, MI) and consist of a hexagonal channel with inlet and outlet connections at the ends of the channel (20mm length, 10mm width, and 200 µm depth) (Fig. 1B,C). The thickness/depth of the microchannel was selected to resemble the diameter of distal pulmonary airways. PDMS was selected due to its excellent stability under physiological-like conditions, biocompatibility, and transparency which allowed imaging inside of the microchannels using regular light and fluorescence microscopy.

The PDMS microfluidic chambers were molded from a silicon master fabricated via standard UV photolithography. Briefly, before spin coating the Si wafers were pre-cleaned with acetone, methanol, and ethanol, and then treated with oxygen plasma (oxygen at 10 sccm, 200 watts, and < 100 mTorr, for 30 seconds), in order to improve the adhesion of the photoresist to the substrate. To further improve the stability of the SU8-2075 layer, an additional thin layer (5 µm) of SU8- 2005 was spin coated (at 3000 RPM for 30 seconds) into the Si wafer and flood exposed prior to the deposition of the negative tone photoresist SU-8 2075 (Microchem Corp, USA). The photoresist SU-8 2075 was then spin coated on a 4 inch silicon (Si) wafer at 1000 rpm for 30 seconds to obtain a ~200–250 µm thick layer. The coated wafer was subsequently pre-baked following the directions provided by the manufacturer, and then exposed to ultraviolet light through the photomask previously developed in SolidWorks, which contains the layout of the hexagonal channel. Finally, the patterned wafer was post-processed following the parameters suggested by the manufacturer.

The PDMS microfluidic chambers were obtained by casting PDMS solutions (1:10 ratio of curing agent to precursor) on top of the patterned silicon wafer. The PDMS solutions were allowed to cure for 24 hours at 60 °C, and then peeled from the silicon master. The thickness of the PDMS microfluidic chambers was confirmed via scanning electron microscopy (SEM) (Fig. 1C).

#### Nanofiber mesh fabrication

Nanofiber meshes were fabricated using a standard electrospinning apparatus which consists of a syringe pump, a high voltage power supply, and a collector. Polymeric nanofibers with different stiffness were obtained by varying the ratio of polycaprolactone – PCL (Mw 14,000, Sigma-Aldrich, USA)/gelatin (porcine skin gelatin type A, Sigma-Aldrich, USA) blends. The polymer blends were produced by mixing PCL and gelatin in hexafluoro-2-propanol – HFP (Sigma-Aldrich, USA) for 24 hours at room temperature. Four PCL/gelatin weight ratios were used in this study: 100/0, 75/25, 50/50 and 25/75. These polymer blends were electrospun at 26 KV at an extrusion rate of 16 mL/h for 2 minutes with a 25 cm distance between the needle tip and collector. These electrospinning parameters were selected in order to obtain ~20 µm thick nanofiber meshes, which resembles the thickness of the lungs basement membrane^43^. The fiber morphology was characterized via SEM and representative SEM images were used to measure the fiber cross-sectional size using the image analysis software Image J (NIH, USA). Tensile test were conducted on dog-bone shaped samples previously immersed in phosphate buffered saline (PBS) for 1 hour, with a cross-head speed of 5 mm/min until failure. The Young’s modulus was calculated as the slope of the most linear region of the stress vs. strain curves prior to the yield point.

#### Assembly of the nano-micro biomimetic system

The three dimensional sealed configuration was obtained by sandwiching the fiber mesh between both microfluidic layers. The binding between microfluidic layers was achieved via oxygen plasma treatment using a TECHNICS Benchtop reactive ion etching at 50 W and 10 cm^3^/min at standard conditions (sccm) O_2_ for 30 seconds. The assembled devices were subsequently heated at 70 °C for 10 min to facilitate PDMS-to-PDMS bonding.

### Cell culture

Human alveolar epithelial cells (A549) (ATCC, Manassas, VA) were regularly maintained in Dulbecco’s Modified Eagle’s Medium (DMEM) (Cellgrowth – Corning, Manassas, VA), supplemented with 10% Fetal Bovine Serum (FBS) (HyClone – Thermo Scientific, Rockford, IL) and 1% of antibiotics/antimycotics mixture (Life Technologies, Grand Island, NY). Human umbilical vein endothelial cells (HUVEC) (ATCC, Manassas, VA) were expanded on gelatin-coated culture flasks, and regularly maintained in Medium 200 prepared without phenol red (Life Technologies, Grand Island, NY), supplemented with low serum growth supplements (LSGS) (Life Technologies, Grand Island, NY) and gentamicin/Amphotericin solutions (Life Technologies, Grand Island, NY) in the concentrations recommended by the manufacturer. Primary human small airway epithelial cells (HSAEC) (PromoCell GmbH, Heidelberg, Germany) were regularly maintained in small airway epithelial cell growth medium (PromoCell GmbH, Heidelberg, Germany) supplemented with a Supplement-Mix (PromoCell GmbH, Heidelberg, Germany). All cell lines were incubated in a humid atmosphere at 37 °C and 5% CO_2_.

After device assembly and prior to cell seeding, all fiber meshes were sterilized using UV light and subsequently coated on both sides using a 0.33 mg/mL growth factor reduced basement membrane matrix solution (BD Biosciences, San Jose, CA) for 1 hour at room temperature. This solution was in serum free Dulbecco’s Modified Eagle Medium/Nutrient Mixture F-12 (DMEM/F-12) (Gibco – Life Technologies, Grand Island, NY).

For co-culture experiments, HUVEC cells were injected into the bottom microfluidic chamber at a cell density of 1.8 × 10^5^ cells/cm^2^, and the device was flipped over to favor gravity-mediated cell settling and subsequent adhesion. After 24 hours, the device was flipped over and a suspension of A549 or HSAEC cells (1.6 × 10^5^ cells/cm^2^) was injected into the top microfluidic chamber. The co-culture was maintained for 48 hours to reach confluence of both cell types. Culture media was changed every 24 hours.

### Evaluation for cellular responses

#### Actin cytoskeleton

Actin distribution was evaluated on confluent cultures via fluorescence and confocal fluorescence microscopy. Briefly, cells were fixed with 10% Neutral Buffered Formalin (Thermo Scientific, Rockford, IL) for 15 minutes and permeabilized with a 0.1% Triton X-100 (Sigma-Aldrich, St. Louis, MO) solution in PBS for 5 minutes. Actin filaments were stained using Alexa Fluor 488 Phalloidin (Invitrogen, Grand Island, NY) (0.1 μM in PBS for 45 minutes). Cell nuclei were stained using DAPI (Sigma-Aldrich, St. Louis, MO) (0.2 μg/mL in PBS for 5 minutes).

#### Tight junction formation

Formation of tight junctions was characterized via immunostaining of cytoplasmic tight junction (TJ) proteins Zonula Occludens-1 (ZO-1) and the TJ transmembrane protein Occludin. For this purpose, cells cultured on the nano-micro biomimetic system were fixed with methanol (Fisher Scientific, USA) at −20 °C for 15 minutes, and permeabilized with a 0.5% Triton X-100 (Sigma-Aldrich, St. Louis MO) solution in PBS for 10 minutes. Non-specific binding was blocked using SuperBlock blocking buffer (Thermo Scientific, Rockford, IL). Subsequently, primary antibodies for anti-ZO-1 (1/100) (Life technologies #40-2200, USA) and anti-occludin (1/250) (Life technologies #33-1500, USA) were applied to the cells at 4 °C overnight. After this time the secondary antibodies (goat anti-mouse Alexa Fluor 488 (1/250) and goat anti-rabbit Alexa Fluor 567 (1/100)) (Invitrogen, Grand Island NY) were added and incubated under dark conditions for 1 hour at room temperature. Cell nuclei was stained using DAPI (4, 6-diamidino-2-phenylindole) (Invitrogen, Grand Island NY) (0.2 µg/ml) for 5 minutes.

TJ were further characterized via western blot analysis. For this analysis, after treatment, the samples were place on ice and the cells were harvested by immersing the fiber meshes into 50 µL of lysis buffer (10 mM HEPES, pH 7.9; 10 mM KCl; 0.1 mM EDTA; 0.1 mM EGTA; 1 mM DTT; 0.5 mM PMSF; 12.8% protease inhibitor cocktail; and 10 µL of 10% Igepal), vortexed for 15 min at 4 °C and placed overnight at −20 °C. The samples were subsequently homogenized using a pellet pestle motor (Fisherbrand, USA) and centrifuged at 17 g for 15 min at 4 °C. After centrifugation, the supernatant was collected as cytosolic lysate.

Protein extracts were resolved in a 4–20% Mini-PROTEAN TGX gel (Bio-Rad, USA) and transfered to a nitrocellulose membrane using a wet transfer system, overnight at 35 V at 4 C. The membranes were blocked with 5% milk for 1 hour at room temperature and probed with antibodies against Occludin (1/500) (Life technologies #71-1500, USA)^44^, ZO-1 (1/500) (Life technologies #40-2200, USA)^45^, GAPDH (1/1000) (Abcam, USA) or actin (1/2000) (Abcam, USA) overnight at 4 °C. The membranes were washed three times with 0.1% tween in TBS and incubated with anti-mouse or anti-rabbit Amersham ECL HRP Conjugated secondary antibodies (1/5000) for 1 hour at room temperature, the washes were repeated and the membranes were developed using a ChemiDoc XRS System (Bio-Rad, USA) and analyzed using the Image Lab Software (Bio-Rad, USA).

#### Epithelial/endothelial barrier function

Changes in barrier function for co-cultured cells on the different fiber meshes were evaluated using a previously described permeability assay^46^. In this technique the diffusion rate of fluorescein isothiocyanate (FITC)-labeled dextran (70 KDa) (Sigma-Aldrich, St. Louis MO) from the top to the bottom microfluidic chamber was measured. First, a 1 mg/mL solution of FITC-dextran was injected into the top airway chamber and a spectro-fluorimeter (BioTek Synergy HT; 485 nm excitation and 528 nm emission) was used to record changes in fluorescence units in the bottom chamber after 60 minutes. An apparent permeability coefficient (P_app_) was calculated according to the following equation: P_app_ = ΔQ/(A*Δt*C_0_), where ΔQ is the change in concentration in the bottom chamber, A is the surface area of the monolayer, Δt is the time of 60 min and C_0_ is the initial concentration in the upper chamber. Note that ΔQ was determined using a calibration curve that relates fluorescent intensity with concentration.

### Fluid-filled airway reopening simulations

As described in our previous studies^15, 16, 22, 28^, airway reopening was simulated by propagating air bubbles over the epithelial monolayer in the top chamber. Briefly, the top microfluidic channel was connected to a programmable PHD 2000 syringe pump (Harvard Apparatus, Holliston, MA) and PBS was injected into the top microfluidic channel, to simulate a surfactant-deficient airway fluid. The fluid was then retracted at a rate of 0.3 mm/s to form an air bubble inside the channel that was propagated across the epithelial cell monolayer (Fig. 1D). Cell injury after 5 reopening simulations was evaluated using a fluorescent live/dead cell viability assay (Life Technologies, Grand Island, NY). The image analysis software Image J (NIH, USA) was used to quantify total number of dead and live cells, and the percentage of cell death was computed as the ratio of live to dead cells after propagation of the air bubbles.

### Statistical analysis

All data were tested for normality using a Shapiro-Wilk test. Data that followed a normal distribution were analyzed using a one-way analysis of variance (ANOVA) with post-hoc least significant difference test and were reported as mean ± standard error. Data that were not normally distributed were analyzed using non-parametric statistical analysis, i.e. ANOVA on ranks with post-hoc Dunn’s test and were reported as median and graphed as median with 75^th^ percentile.

## Electronic supplementary material

Supplement 1

## References

[1] Fan E, Villar J, Slutsky AS (2013). Novel approaches to minimize ventilator-induced lung injury. BMC medicine.

[2] Belperio JA, Keane MP, Lynch JP, Strieter RM (2006). The role of cytokines during the pathogenesis of ventilator-associated and ventilator-induced lung injury. Seminars in respiratory and critical care medicine.

[3] Cavanaugh KJJ, Oswari J, Margulies SS (2001). Role of stretch on tight junction structure in alveolar epithelial cells. Am J Respir Cell Mol Biol.

[4] Tschumperlin DJ, Oswari J, Margulies AS (2000). Deformation-induced injury of alveolar epithelial cells. Effect of frequency, duration, and amplitude. American journal of respiratory and critical care medicine.

[5] Vlahakis NE, Schroeder MA, Limper AH, Hubmayr RD (1999). Stretch induces cytokine release by alveolar epithelial cells *in vitro*. Am J Physiol.

[6] Huang Y, Crawford M, Higuita-Castro N, Nana-Sinkam P, Ghadiali SN (2012). miR-146a regulates mechanotransduction and pressure-induced inflammation in small airway epithelium. FASEB J.

[7] Hong CM (2010). Low tidal volume and high positive end-expiratory pressure mechanical ventilation results in increased inflammation and ventilator-associated lung injury in normal lungs. Anesthesia and analgesia.

[8] Slutsky AS (2005). Ventilator-Induced Lung Injury: From Barotrauma to Biotrauma. Respiratory Care.

[9] Ghadiali SN, Gaver DP (2008). Biomechanics of liquid-epithelium interactions in pulmonary airways. Respir Physiol Neurobiol.

[10] Guldner A (2016). Comparative Effects of Volutrauma and Atelectrauma on Lung Inflammation in Experimental Acute Respiratory Distress Syndrome. Critical care medicine.

[11] Douville NJ (2011). Combination of fluid and solid mechanical stresses contribute to cell death and detachment in a microfluidic alveolar model. Lab Chip.

[12] Hussein O (2013). Biophysical determinants of alveolar epithelial plasma membrane wounding associated with mechanical ventilation. American journal of physiology. Lung cellular and molecular physiology.

[13] Bilek AM, Dee KC, Gaver DP (2003). Mechanisms of surface-tension-induced epithelial cell damage in a model of pulmonary airway reopening. J Appl Physiol.

[14] Kay SS, Bilek AM, Dee KC, Gaver DP (2004). Pressure gradient, not exposure duration, determines the extent of epithelial cell damage in a model of pulmonary airway reopening. J Appl Physiol.

[15] Yalcin HC, Perry SF, Ghadiali SN (2007). Influence of Airway Diameter and Cell Confluence on Epithelial Cell Injury in an *In-Vitro* Model of Airway Reopening. Journal of Applied Physiology.

[16] Yalcin HC (2009). Influence of cytoskeletal structure and mechanics on epithelial cell injury during cyclic airway reopening. American journal of physiology. Lung cellular and molecular physiology.

[17] Vlahakis NE, Hubmayr RD (2000). Invited Review: Plasma membrane stress failure in alveolar epithelial cells. J Appl Physiol.

[18] Birukov KG (2002). Shear stress-mediated cytoskeletal remodeling and cortactin translocation in pulmonary endothelial cells. Am J Respir Cell Mol Biol.

[19] Birukov KG (2003). Magnitude-dependent regulation of pulmonary endothelial cell barrier function by cyclic stretch. American journal of physiology. Lung cellular and molecular physiology.

[20] Edwards YS (2001). Stretch stimulation: its effects on alveolar type II cell function in the lung. Comp Biochem Physiol A Mol Integr Physiol.

[21] Tschumperlin DJ, Margulies SS (1998). Equibiaxial deformation-induced injury of alveolar epithelial cells *in vitro*. Am J Physiol.

[22] Higuita-Castro N, Shukla VC, Mihai C, Ghadiali SN (2016). Simvastatin Treatment Modulates Mechanically-Induced Injury and Inflammation in Respiratory Epithelial Cells. Ann Biomed Eng.

[23] Vlahakis NE, Hubmayr RD (2005). Cellular stress failure in ventilator-injured lungs. American journal of respiratory and critical care medicine.

[24] Vlahakis NE, Schroeder MA, Pagano RE, Hubmayr RD (2002). Role of Deformation-induced Lipid Trafficking in the Prevention of Plasma Membrane Stress Failure. American journal of respiratory and critical care medicine.

[25] Huh D (2010). Reconstituting organ-level lung functions on a chip. Science.

[26] Benam KH (2016). Small airway-on-a-chip enables analysis of human lung inflammation and drug responses *in vitro*. Nat Methods.

[27] Punde TH (2015). A biologically inspired lung-on-a-chip device for the study of protein-induced lung inflammation. Integr Biol (Camb).

[28] Higuita-Castro N, Mihai C, Hansford DJ, Ghadiali SN (2014). Influence of airway wall compliance on epithelial cell injury and adhesion during interfacial flows. J Appl Physiol.

[29] Schindler M (2005). A synthetic nanofibrillar matrix promotes *in vivo*-like organization and morphogenesis for cells in culture. Biomaterials.

[30] Lim SH, Mao HQ (2009). Electrospun scaffolds for stem cell engineering. Advanced drug delivery reviews.

[31] Nisbet DR, Forsythe JS, Shen W, Finkelstein DI, Horne MK (2009). Review paper: a review of the cellular response on electrospun nanofibers for tissue engineering. Journal of biomaterials applications.

[32] Nur EKA, Ahmed I, Kamal J, Schindler M, Meiners S (2006). Three-dimensional nanofibrillar surfaces promote self-renewal in mouse embryonic stem cells. Stem cells.

[33] Zhao M (2010). The three-dimensional nanofiber scaffold culture condition improves viability and function of islets. Journal of biomedical materials research. Part A.

[34] Liu, F. & Tschumperlin, D. J. Micro-mechanical characterization of lung tissue using atomic force microscopy. *J Vis Exp* (2011). 10.3791/2911 PMC3217627 21897356

[35] Wysolmerski RB, Lagunoff D (1990). Involvement of myosin light-chain kinase in endothelial cell retraction. Proc Nati Acad Sci USA.

[36] Garcia JGN, Davis HW, Patterson CE (1995). Regulation of Endothelial Cell Gap Formation and Barrier Dysfunction: Role of Myosin Light Chain Phosphorylation. J Cell Physiol.

[37] Parker JC (2000). Inhibitors of myosin light chain kinase and phosphodiesterase reduce ventilator-induced lung injury. J Appl Physiol.

[38] Jacob, A. M. & Gaver, D. P. An investigation of the influence of cell topography on epithelial mechanical stresses during pulmonary airway reopening. *Physics of Fluids* **17** (2005). 10.1063/1.1862642 PMC3672399 23745044

[39] Jacob AM, Gaver DP (2012). Atelectrauma disrupts pulmonary epithelial barrier integrity and alters the distribution of tight junction proteins ZO-1 and claudin 4. Journal of applied physiology (Bethesda, Md.: 1985).

[40] Dailey HL, Ghadiali SN (2010). Influence of power-law rheology on cell injury during microbubble flows. Biomech Model Mechanobiol.

[41] Dailey HL, Yalcin HC, Ghadiali SN (2007). Fluid-structure modeling of flow-induced alveolar epithelial cell deformation. Computers & Structures.

[42] Li M (2005). Electrospun protein fibers as matrices for tissue engineering. Biomaterials.

[43] Brightling CE (2002). Mast-cell infiltration of airway smooth muscle in asthma. N Engl J Med.

[44] Stammler A (2016). Highly Conserved Testicular Localization of Claudin-11 in Normal and Impaired Spermatogenesis. PLoS One.

[45] Guo YH (2016). Wnt/β-catenin pathway transactivates microRNA-150 that promotes EMT of colorectal cancer cells by suppressing CREB signaling. Oncotarget.

[46] Huang Y, Haas C, Ghadiali SN (2010). Influene of Transmural Pressure and Cytoskeletal Structure on NF-KB Activation in Respiratory Epithelial Cell. Cellular and Molecular Bionengineering.

