# Supplementary material for: Using a Novel Microfabricated Model of the Alveolar-Capillary Barrier to Investigate the Effect of Matrix Structure on Atelectrauma
Source: Sci Rep. 2017 Sep 14;7:11623. doi: 10.1038/s41598-017-12044-9 (PMC5599538; doi:10.1038/s41598-017-12044-9)

Supplemental Information

Manuscript Title: *Using a Novel Microfabricated Model of the Alveolar-Capillary Barrier to Investigate the Effect of Matrix Structure on Atelectrauma*

Author List:
N. Higuita-Castro,[1,2] M.T. Nelson,[1] V. Shukla,[1,2] P.A. Agudelo-Garcia,[3] W. Zhang,[4] S.M. Duarte-Sanmiguel,[1,5] J.A. Englert[4] J.J. Lannutti,[6] D.J. Hansford,[1] and S.N. Ghadiali[1,2,4]*

[1]Biomedical Engineering Department, The Ohio State University, Columbus, Ohio
[2]Dorothy M. Davis Heart and Lung Research Institute, The Ohio State University Wexner Medical Center, Columbus, Ohio
[3]Department of Molecular and Cellular Biochemistry, The Ohio State University, Columbus, Ohio
[4]Department of Internal Medicine, Division of Pulmonary, Critical Care and Sleep Medicine, The Ohio State University Wexner Medical Center, Columbus, Ohio
[5]Human Nutrition Program, The Ohio State University, Columbus, Ohio
[6]Department of Material Sciences and Engineering, The Ohio State University, Columbus, Ohio

A549_ZO-1 Expression

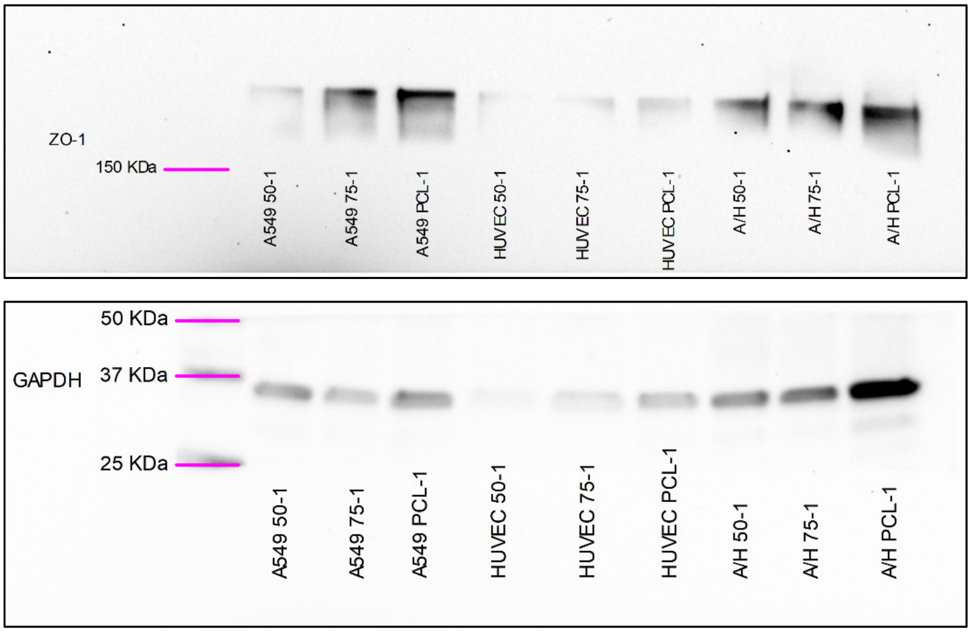

A549_OCLN Expression

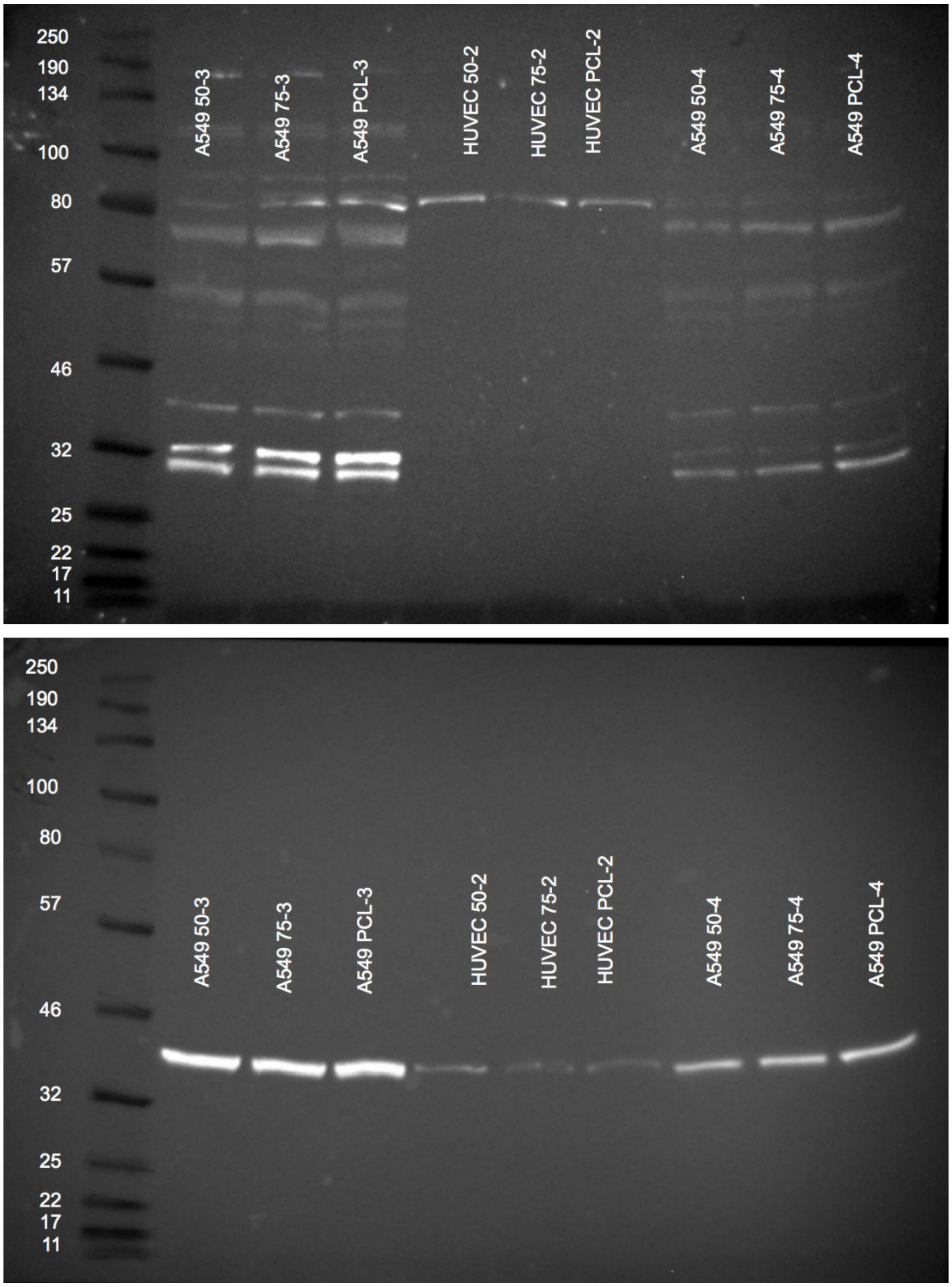

HUVEC_ZO-1 and OCLN Expression

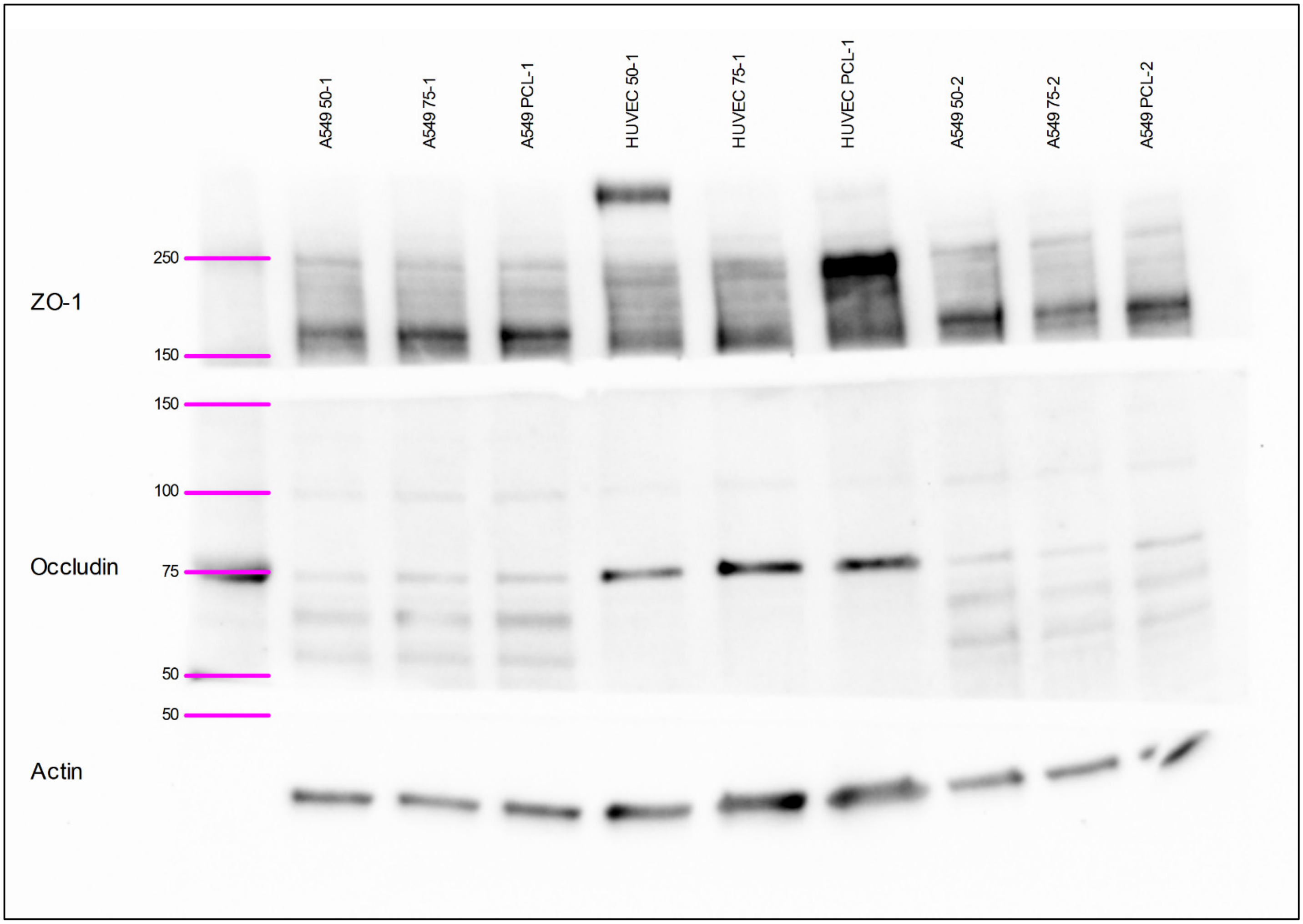

Supplement: Supplementary file 1 — Supplement 1 [file 41598_2017_12044_MOESM1_ESM.pdf]
